# Care by general practitioners for patients with asthma or COPD during the COVID-19 pandemic

**DOI:** 10.1038/s41533-023-00340-z

**Published:** 2023-04-08

**Authors:** Corinne Rijpkema, Lotte Ramerman, Maarten Homburg, Eline Meijer, Jean Muris, Tim olde Hartman, Marjolein Berger, Lilian Peters, Robert Verheij

**Affiliations:** 1 Nivel, Netherlands Institute for Health Services Research, Utrecht, The Netherlands; 2 Tilburg School of Social and Behavioural Sciences, Tilburg University, Tilburg, The Netherlands; 3 Department of General Practice and Elderly Care Medicine, UMCG, University Medical Centre Groningen, Groningen, The Netherlands; 4 Data Science Centre in Health (DASH), UMCG, University Medical Centre Groningen, Groningen, The Netherlands; 5 Department of Family Medicine, CAPHRI Care and Public Health Research Institute, Maastricht University, Maastricht, The Netherlands; 6 Radboud Institute of Health Sciences, Department of Primary and Community Care, Radboud University Nijmegen Medical Centre, Nijmegen, The Netherlands; 7 Vrije Universiteit Amsterdam, Midwifery Science, AVAG, Amsterdam Public Health, Amsterdam University Medical Centre, Amsterdam, The Netherlands

**Keywords:** Health care, Asthma, Chronic obstructive pulmonary disease

## Abstract

The impact of the COVID-19 pandemic on general practitioners’ (GP) care for patients with asthma and/or COPD is largely unknown. To describe the impact of the pandemic on asthma or COPD-related GP care, we analysed routinely recorded electronic health records data from Dutch general practices and out-of-hours (OOH) services. During the COVID-19 pandemic (2020), the contact rates for asthma and/or COPD were significantly lower in GP practices and OOH services compared with the pre-pandemic period (2019) (respectively, 15% lower and 28% lower). The proportion of telephone contacts increased significantly with 13%-point in GP practices and 12%-point at OOH services, while the proportion of face-to-face contacts decreased. Furthermore, the proportion of high urgent contacts with OOH services decreased by 8.5%-point. To conclude, the overall contact rates in GP practices and OOH services decreased, while more contacts were remote. Lower contact rates have, after a short follow-up, not resulted in more patients with exacerbations in OOH care. However, this might still be expected after a longer follow-up.

## Introduction

The COVID-19 pandemic has had an enormous impact on public health and health care. In the first year of the pandemic, there were ~5 million reported infections and ~90,000 COVID-related reported deaths worldwide, of which 1.7 million and 35,000 were in Europe^1^. However, not everyone is equally affected by the COVID-19 pandemic^2^. Particularly, patients with (chronic) comorbidity were more likely to have a more severe course of their disease from a COVID-19 infection^3–6^. This may lead, for example for patients with asthma or Chronic Obstructive Pulmonary Disease (COPD), to more exacerbations and structural damage in the lungs, worsening their respiratory condition^7^. Measures to prevent the spread of the virus also affected these patients indirectly, as regular care with their general practitioner (GP), including disease management programmes for chronically ill patients, were postponed (e.g. lung function tests and consultations) or provided remotely (e.g. by telephone, video or e-consult)^8–12^. Furthermore, many chronically ill patients did not visit their GP during the COVID-19 pandemic because they were afraid of becoming infected with SARS-CoV-2^13^.

In the Netherlands, GPs are the first point of contact for patients and are the gatekeepers to specialised secondary care^14^ (Box 1). In addition, GPs and practice nurses (a nurse who works in a GP office) play an important role in the care and management of patients with chronic diseases, such as asthma or COPD^14^. In 2020, ~1.1 million Dutch people (of a total population of ~17.4 million) had asthma and/or COPD^15,16^. These patients consult their GP and practice nurse regularly as part of disease management programmes e.g. to assess their burden of illness and discuss lifestyle and (inhaled) medication. Furthermore, the GP can refer patients to other healthcare providers if indicated^17^. Regular check-ups and consultations are meant to reduce symptoms and prevent exacerbations^18–20^. As a consequence, when this regular care is suspended, postponed, or avoided, patients are expected to have more exacerbations of their condition, needing immediate care, including out-of-hours. Therefore, OOH services and other emergency care providers act as a safety net throughout the health system and can be an indicator of problems caused by changes elsewhere in the health system^21,22^.

The COVID-19 pandemic and associated measures may both have had an impact on the healthcare use of patients with asthma and/or COPD. However, it is unclear what the impact is of the COVID-19 pandemic on asthma and/or COPD-related care. Therefore, this study aimed to describe the impact of the COVID-19 pandemic on asthma or COPD-related care from GP practices and OOH services. We aimed to answer the following research questions: (1) How did contact rates for patients with asthma and COPD in GP practices and OOH services differ during various phases of the COVID-19 pandemic compared to 2019? (2) How did these contacts take place during the phases of the COVID-19 pandemic compared to 2019? and (3) To what extent did the urgency of asthma and COPD contacts at the OOH services change during the COVID-19 pandemic compared to 2019?

Box 1. General practice care in the Netherlands General practitioners (GPs) are the first point of contact for patients with a healthcare professional. Dutch GPs are the gatekeepers for specialised secondary care (referral system)^14^ and virtually every citizen is listed as a patient in a specific practice (list system). During office hours, GP care is provided in local general practices with one or more GPs and practice nurses. Outside office hours, GP care is provided in regional out-of-hours (OOH) services in central locations (often in conjunction with a hospital) populated by GPs and triagists who assess levels of urgency and determine the follow-up action.

*General practices*

GPs assess patients’ physical and mental symptoms, problems, and urgency, taking into account the medical history, and preferences of the patient^14^. Together with the patient, GPs determine which care is necessary and provide this care or refer to other healthcare professionals. GPs are also responsible for preventative care of chronic patients (diabetes, COPD, and cardiovascular risk management) to avert complications. In addition, they provide preventive care for mental health problems and older adults to support physical, cognitive, and psychological frailty^14^. In GP practices, most contacts are face-to-face.

*Out-of-hours services*

During the evening, nights, and weekends, OOH services provide urgent medical care, which must be evaluated immediately or within a few hours^14^. Prior to a contact with the OOH service, patients should first call the OOH services, where the telephone triagist assesses the level of urgency based on the severity of the complaints stated by the patient (U0 loss of vital functions – U5 no chance of harm)^47^. The level of urgency determines how quickly a patient will receive care and whether this will be through telephone contact, contact at the OOH service location, or via home visit^47^.

## Results

Table 1 provides an overview of the characteristics of the patient populations with contact(s) for asthma and/or COPD in GP practices (during office hours) and at OOH services.

**Table 1:** Characteristics of the patient populations in the databases for GP practices and OOH services.

	GP practices		OOH services	
	2019	2020	2019	2020
Number of patients (with at least one contact) per 1000 registered patients/inhabitants of catchment area				
Asthma	26.9	23.0	1.1	0.8
COPD	16.0	13.8	0.9	0.7
Number of contacts per 1000 registered patients/inhabitants of catchment area				
Asthma	68.6	57.3	1.2	0.9
COPD	59.8	50.9	1.2	0.9
Sex in %				
Male	44.4	43.5	46.2	46.7
Female	55.6	56.5	53.8	53.3
Age in %				
0–4 years	1.6	1.0	8.5	5.1
5–17 years	6.5	5.6	9.5	10.1
18–44 years	19.4	20.7	19.0	22.0
45–69 years	45.5	45.3	31.7	32.6
70 years and older	27.0	27.4	31.3	30.2

### Contact rates for asthma or COPD in 2020 (during the COVID-19 pandemic) compared to 2019

**Fig. 1:**
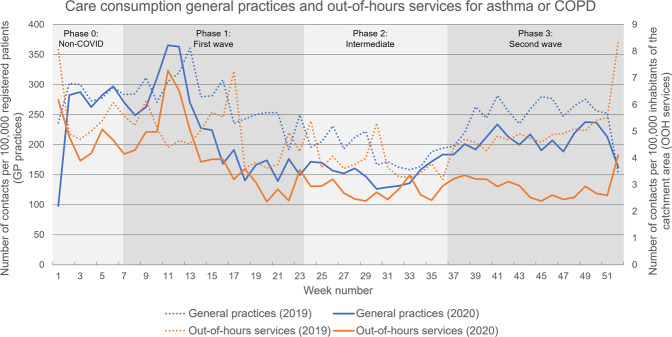
The contact rates for asthma or COPD in general practices and out-of-hours services. The contact rates for asthma or COPD in general practices (blue) and out-of-hours services (orange), for 2019 (dots) and 2020 (lines).

The overall contact rates for asthma or COPD-related care in general practices and at OOH services were lower in 2020 (during the COVID-19 pandemic) compared to 2019. In 2019, there were 127.9 contacts for asthma or COPD per 1000 registered patients in GP practices, compared to 108.6 contacts per 1000 in 2020 (Table 1). This represents a decrease of 15%. After an initial increase in contacts for asthma or COPD in GP practices at the start of the pandemic (weeks 9–13), contact rates decreased considerably, resulting in a lower contact rate during phase 1 in 2020, than in the same period in 2019 (Fig. 1 and Table 2). However, due to the fluctuation in this period, phase 1 did not significantly differ from the same period in 2019 (*p* = 0.081). In the second and third phases of the COVID-19 pandemic, the contact rates in GP practices were significantly lower in 2020 than in 2019 (resp. *p* = 0.001 and *p* < 0.001).

**Table 2:** Mean and standard deviation for the contact rates, the proportion of the type of contact (2019 and 2020), and differences in contact rates and the type of contact between 2019 and 2020 presented per phase of the COVID-19 pandemic, both for GP practices and OOH services.

	2019	2020	Difference between 2019 and 2020			
	Mean (SD)	Mean (SD)	Coefficient	95% CI		*p* value
GP practices contacts per 100,000 registered patients						
Phase 0	281.7 (21.1)	253.8 (64.8)	−28.0	−72.6	16.7	0.220
Phase 1	269.4 (45.0)	218.9 (75.3)	−50.5	−107.2	6.2	0.081
Phase 2	189.7 (23.9)	154.4 (19.7)	−35.3	−56.5	−14.2	0.001
Phase 3	251.0 (32.3)	208.4 (20.4)	−42.6	−62.7	−22.5	<0.001
OOH services contacts per 100,000 inhabitants of the catchment area						
Phase 0	5.6 (1.1)	4.7 (0.7)	−1.0	−1.8	−0.1	0.031
Phase 1	4.9 (1.0)	4.0 (1.4)	−0.8	−1.9	0.2	0.127
Phase 2	3.8 (0.6)	2.8 (0.3)	−1.0	−1.4	−0.6	<0.001
Phase 3	5.1 (0.9)	2.9 (0.4)	−2.1	−2.7	−1.6	<0.001
The proportion of the type of contact in GP practices^a^	% (SD)	% (SD)	Coefficient	95% CI		*p* value
Face-to-face contact						
Phase 0	75.4% (0.7%)	73.0% (0.9%)	−0.1	−0.2	−0.1	<0.001
Phase 1	75.4% (2.1%)	57.5% (7.6%)	−0.8	−1.0	−0.6	<0.001
Phase 2	75.0% (1.2%)	62.1% (2.5%)	−0.6	−0.7	−0.5	<0.001
Phase 3	75.3% (1.3%)	63.2% (3.4%)	−0.6	−0.7	−0.5	<0.001
Telephone contact						
Phase 0	15.7% (9.4%)	17.7% (1.4%)	0.1	0.1	0.2	<0.001
Phase 1	16.7% (1.6%)	36.3% (9.2%)	1.0	0.8	1.3	<0.001
Phase 2	17.4% (1.2%)	30.0% (3.2%)	0.7	0.6	0.8	<0.001
Phase 3	16.3% (1.3%)	30.5% (3.0%)	0.8	0.7	0.9	<0.001
Home visits						
Phase 0	8.7% (1.0%)	8.9% (1.3%)	0.1	−0.1	0.2	0.649
Phase 1	7.7% (1.2%)	5.4% (1.9%)	−0.4	−0.6	−0.1	0.003
Phase 2	7.4% (1.2%)	7.3% (1.7%)	−0.1	−0.2	0.2	0.933
Phase 3	8.0% (1.3%)	5.7% (0.8%)	−0.4	−0.5	−0.2	<0.001
The proportion of the type of contact in OOH services						
Face-to-face contact						
Phase 0	48.8% (1.2%)	47.4% (2.0%)	−0.1	−0.1	0.1	0.066
Phase 1	51.1% (3.1%)	39.0% (6.3%)	−0.5	−0.6	−0.3	<0.001
Phase 2	48.8% (3.7%)	40.2% (4.7%)	−0.3	−0.5	−0.2	<0.001
Phase 3	52.7% (2.5%)	37.8% (4.5%)	−0.6	−0.7	−0.5	<0.001
Telephone contact						
Phase 0	18.7% (1.1%)	19.9% (0.9%)	0.1	0.1	0.1	0.032
Phase 1	21.0% (2.8%)	35.4% (6.3%)	0.7	0.5	0.9	<0.001
Phase 2	23.8% (3.0%)	34.5% (3.5%)	0.5	0.4	0.7	<0.001
Phase 3	20.1% (2.0%)	35.8% (2.1%)	0.8	0.7	0.9	<0.001
Home visits						
Phase 0	32.5% (1.8%)	32.6% (1.5%)	0.1	−0.1	0.1	0.977
Phase 1	28.0% (3.4%)	25.7% (4.0%)	−0.1	−0.3	0.3	0.124
Phase 2	27.5% (2.8%)	25.3% (3.3%)	−0.1	−0.2	0.1	0.081
Phase 3	27.2% (2.4%)	26.4% (3.5%)	−0.1	−0.2	0.1	0.472

^a^The proportion of the type of contacts in GP practices does not add up to 100%, because digital consultations are not included in this table.

In 2019, there were 2.5 contacts per 1000 inhabitants of the catchment area for asthma and/or COPD with OOH services, compared to 1.8 contacts per 1000 in 2020 (Table 1). This represents a decrease of 28%. During the first wave of the COVID-19 pandemic, there was a steep increase in contacts for asthma or COPD with OOH services between weeks 11 and 14 (Fig. 1). After this initial increase, contact rates in phase 1 decreased considerably and remained lowered. Due to this fluctuation, phase 1 did not significantly differ from the same period in 2019 (*p* = 0.127) (Table 2). During phases 2 and 3, the contact rates were significantly lower in 2020 for asthma/COPD, than in the same periods in 2019 at OOH services (both *p* < 0.001).

### Type of contact for asthma or COPD in 2020 (during the COVID-19 pandemic) compared to 2019

**Fig. 2:**
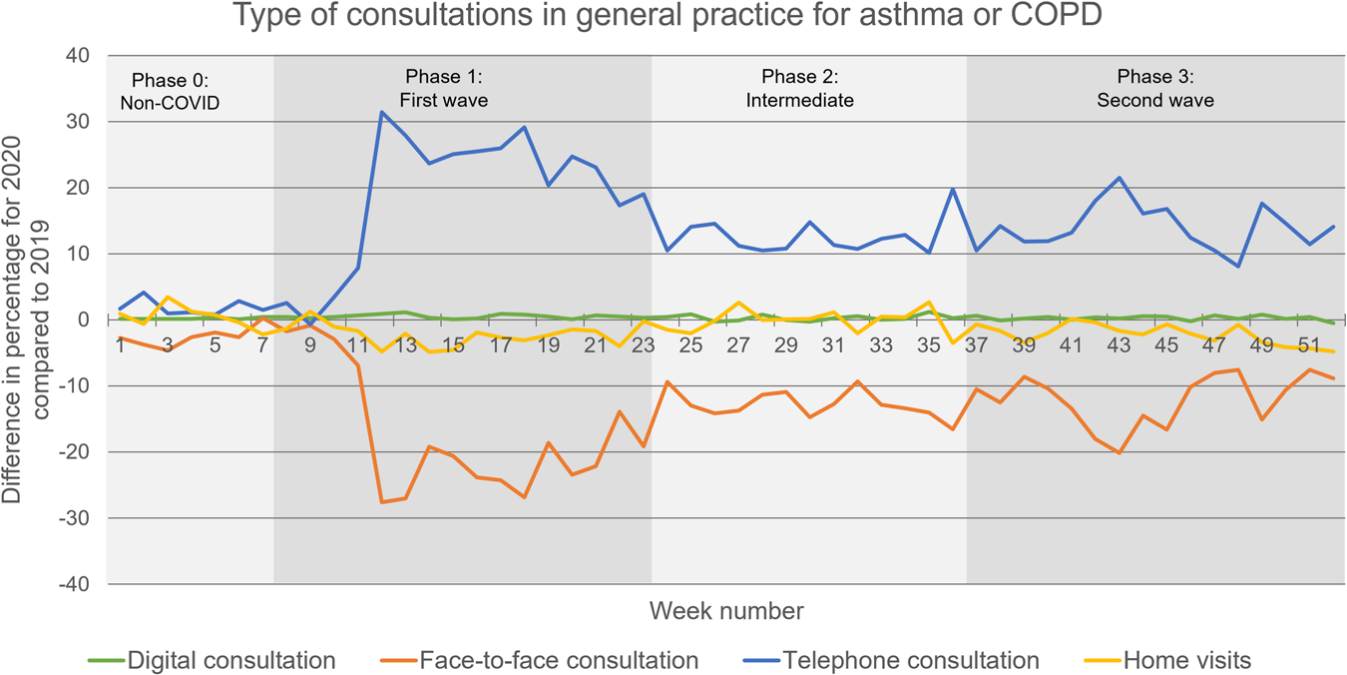
Type of consultations in general practice for asthma or COPD. The difference in type of consultation in general practice for asthma or COPD, 2020 compared to 2019.

During the COVID-19 pandemic, there was a shift from face-to-face contacts to telephone contacts for asthma and COPD-related care. The proportion of face-to-face contacts in GP practices significantly decreased from 75% in 2019 to 63% in 2020 (all phases *p* < 0.001), while the proportion of telephone contacts significantly increased from 17% in 2019 to 30% in 2020 (all phases *p* < 0.001), see Fig. 2 and Table 2. The decrease in the proportion of face-to-face contacts and the increase in the proportion of telephone contacts was initiated in phase 1 and partly reversed in the following phases (Fig. 2). The proportion of home visits decreased from 8% in 2019 to 6% in 2020, with a significant decrease in phases 1 (*p* = 0.003) and 3 (*p* < 0.001) during the pandemic, compared to 2019 (Table 2).

**Fig. 3:**
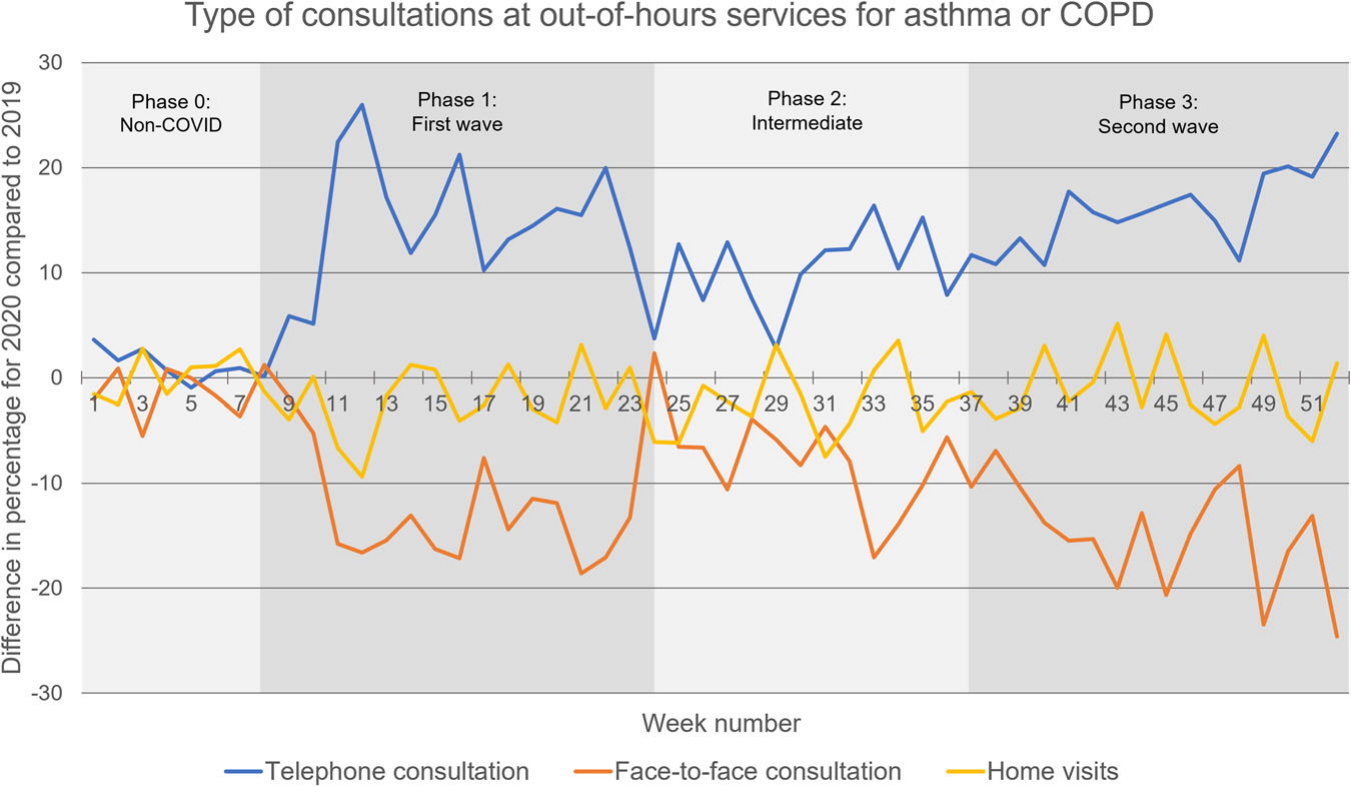
Type of consultations at out-of-hours services for asthma or COPD. The difference in type of consultation at out-of-hours services for asthma or COPD, 2020 compared to 2019.

The proportion of face-to-face contacts at OOH services decreased significantly from 51% in 2019 to 40% in 2020 (phase 0: *p* = 0.066, phases 1–3: *p* < 0.001), while the proportion of telephone contacts significantly increased from 21% in 2019 to 33% in 2020 (phase 0: *p* = 0.032, phases 1–3: *p* < 0.001), see Fig. 3 and Table 2. The proportion of home visits did not change significantly in 2020 compared to 2019.

### Changes in the type of contact for asthma or COPD during the various phases of the COVID-19 pandemic

When comparing the various phases in 2020 (during the COVID-19 pandemic) in GP practices, the proportion of face-to-face contacts was significantly lower in phase 1 compared to phase 0, while the proportion of telephone contacts was significantly higher (both *p* < 0.001), see Fig. 2 and Table 3. However, the proportion of telephone contacts again became significantly lower in phase 2 compared to phase 1, while face-to-face contacts became significantly higher (respectively, *p* = 0.028 and *p* = 0.015). During the COVID-19 pandemic, the proportion of home visits only decreased significantly in phase 1 compared to phase 0 (*p* < 0.001), see Table 3.

**Table 3:** Differences between the phases in 2020 for the type of contact, both for GP practices and OOH services.

	*F*-value	Degrees of freedom	*p* value
Type of contacts GP practices			
Face-to-face contacts			
Phases 0–1	−15.5	51	<0.001
Phases 1–2	5.0		0.028
Phases 2–3	0.6		1.000
Telephone contacts			
Phases 0–1	18.6		<0.001
Phases 1–2	−6.3		0.015
Phases 2–3	0.6		1.000
Home visits			
Phases 0–1	−3.5		<0.001
Phases 1–2	1.5		0.056
Phases 2–3	−1.2		0.312
Type of contacts OOH services			
Face-to-face contacts			
Phases 0–1	−8.5	51	0.001
Phases 1–2	1.4		1.000
Phases 2–3	−3.8		0.296
Telephone contacts			
Phases 0–1	15.5		<0.001
Phases 1–2	−0.9		1.000
Phases 2–3	1.8		1.000
Home visits			
Phases 0–1	−6.9		<0.001
Phases 1–2	−0.5		1.000
Phases 2–3	1.9		0.837

For OOH services, the proportion of telephone contacts significantly increased (*p* < 0.001), while the proportion of face-to-face contacts and the proportion of home visits significantly decreased (respectively, *p* = 0.001 and *p* < 0.001) in phase 1 of the COVID-19 pandemic, compared to phase 0. In phases 2 and 3 of the pandemic, there were no significant changes in the proportions of the different types of care (between phases 1–2 and 2–3) (Table 3).

### Allocation of urgency levels at OOH services

During the COVID-19 pandemic, higher urgency levels were assigned less often to patients who contacted the OOH service for asthma or COPD (Table 4). In 2020, U2 and U3 (very urgent) were assigned less often, compared to 2019, respectively 9.7 to 5.6 per 1000 inhabitants of the catchment area (U2) and 6.2 to 4.6 per 1000 (U3). In contrast, the low urgency (U4 and U5) hardly changed. When looking at the proportional distribution, the urgency level of U2 decreased significantly in 2020 by 8.5%-point, while U4 and U5 significantly increased by 2.6%-point and 5.9%-point respectively (all *p* < 0.001).

**Table 4:** The distribution of urgency levels at OOH services for asthma/COPD, displayed per 1000 inhabitants of the catchment area and the proportion of the distribution, for phases 1–3 in 2019 (before the COVID-19 pandemic) and phases 1–3 in 2020 (during the COVID-19 pandemic).

Urgency level^a^	2019 Pre-pandemic		2020 Pandemic		*Z* (*p* value)^b^
	Per 1000 inhabitants of the catchment area	%	Per 1000 inhabitants of the catchment area	%	
U1 (immediate danger to life—immediate care)	0.6	2.9%	0.4	2.6%	−1.81 (*p* = 0.070)
U2 (threat to vital signs or organ damage—care as soon as possible)	9.7	47.2%	5.6	38.7%	−17.97 (*p* < 0.001)
U3 (real chance of damage—care within a few hours)	6.2	29.9%	4.6	30.2%	2.23 (*p* = 0.026)
U4 (negligible chance of damage—care same day)	1.8	8.9%	1.7	11.5%	8.39 (*p* < 0.001)
U5 (no chance of damage—care next working day)	2.3	11.1%	2.4	17.0%	16.65 (*p* < 0.001)

^a^U0 was not assigned and, therefore, excluded from the table.

^b^Differences between 2019 and 2020 (starting phase 1) in the proportion of the different urgency levels.

### Post hoc analyses

To investigate whether there were differences in contact rates between patients with asthma or COPD, we analysed these separately in a sensitivity analysis for both GP practices and OOH services. No major differences between the two conditions were found. Contact rates in GP practices for patients with asthma decreased by 16.5% in 2020 compared to a decrease of 14.9% for COPD (Table 1). Contact rates with OOH services decreased by 25% for both asthma and COPD (Table 1). In addition, for phases 1, 2, and 3, results were overall similar for asthma and COPD. However, there were some baseline differences (phase 0) between conditions as patients with COPD in GP practices showed a borderline significant decrease (*p* = 0.050) in 2020 vs. 2019 compared to patients with asthma (*p* = 0.507). In OOH services, patients with asthma showed a significant decrease (*p* = 0.013) in 2020 vs. 2019, while COPD did not (*p* = 0.073). As our analyses focused on phases 1, 2 and 3 of the COVID-19 pandemic, this baselined difference was considered not to be relevant, justifying our approach to analyse the two diseases together.

We also performed analyses of the proportional difference in contact rates in 2020 compared to 2019 for different age categories (0–17 years, 18–69 years, 70 years and older). COPD was not included in the analyses for 0–17 years for GP practices and OOH services, because there were no contacts for that age group for COPD. During the start of the COVID-19 pandemic (weeks 9–13) in 2020 compared to 2019, there was an increase in contact rates with GP practices for all age groups, after which contact rates declined for all age groups (from week 13 onwards), see Fig. 1 in Supplementary File. However, for asthma, we observed a greater decrease in contacts for patients aged 0–17 years in GP practices. For OOH services, there was also an increase in contact rates during the start of the COVID-19 pandemic (weeks 10–14) in 2020 compared to 2019, however, only for patients aged 0–17 years and 18–69 years, see Fig. 2 in Supplementary File.

## Discussion

This study showed the impact of the COVID-19 pandemic on general practitioner care for patients with asthma and COPD, both in GP practices (during office hours) and at OOH services, in terms of contact rates, how the care was provided, and the urgency levels of contacts with OOH services. Both in GP practices and at OOH services, contact rates for asthma or COPD decreased during the COVID-19 pandemic. In addition, more care was provided by telephone. In OOH services, the proportion of telephone contacts remained at an increased level during all phases of the COVID-19 pandemic, while in GP practices, the proportion decreased again during a later phase of the pandemic. Furthermore, during the pandemic, higher urgency levels were less often assigned to patients for contacts with OOH services for asthma or COPD.

From the start of the COVID-19 pandemic, a considerable decrease in contact rates for asthma or COPD was observed in GP practices and OOH services. Firstly, the decrease in contact rates in GP practices was likely initiated by the recommendations of ‘The Dutch College of General Practitioners’ (NHG) to delay routine care for patients with asthma or COPD and to suspend regular lung function tests (spirometry). The reduction in chronic care contacts was also observed in Belgium^23^. Secondly, reduced contact rates in both GP practices and OOH services may be explained by fewer exacerbations, as was found by Shah et al.^7^ for asthma patients^7^. The presentation of fewer exacerbations in asthma and COPD patients in both GP practices and OOH services may be related to a decreased circulation of respiratory viruses due to the containment measurements (i.e. social distancing, face masks)^7,24^ and a decrease in air pollution, due to less traffic^25–27^. Thirdly, some patients did not consider their complaints serious enough to make an appointment with their GP, and for other patients, doctors’ assistants have considered this. Patients’ decisions were also influenced by media reports of overcrowded healthcare facilities and they thought that it was not even possible to make an appointment with their GP^28^. Last, it is possible that patients with asthma or COPD improved their self-management skills, due to concerns about getting infected with SARS-CoV-2 when visiting a GP, resulting in a decreased need for care^24,26^. However, it remains unclear to what extent each of the above reasons played a role in the reduction of contact rates in both GP practices and OOH services.

During the COVID-19 pandemic, we observed a relative increase in telephone contacts and a decrease in face-to-face contacts for asthma or COPD-related GP care, which was in line with previous studies^9,13,29^. After the first wave of COVID-19 infections, the proportion of telephone contacts remained heightened in OOH services, while GP practices increased their face-to-face contacts. A possible explanation could be that GPs in GP practices wished to see their patients face-to-face again. In contrast, GPs in OOH services became accustomed to providing care remotely (i.e., telephone contacts). Furthermore, the transition to remote care at OOH services may have resulted in more efficient care and less workload and should be considered as a possible solution to the staffing shortages and high workloads in OOH services^30^. Several studies show that remote contacts for respiratory diseases have potential benefits for access to and effectiveness of care when fully integrated with face-to-face contacts^31–34^. A study into the differences between remote and face-to-face check-ups for asthma showed no significant effects with regard to exacerbations or quality of life^35^. This can be a first step towards the integration of remote care for asthma or COPD patients in the Netherlands. However, when implementing this, the lack of non-verbal communication when using remote care should be taken into account^36^.

Moreover, in this study, we demonstrated that (face-to-face) care for asthma or COPD in general practices was partially suspended during the COVID-19 pandemic. A possible consequence could be that patients with asthma or COPD are less in control of their disease and, therefore, more likely to contact OOH services in case of acute exacerbations of symptoms, as OOH services are seen as a safety net in the whole healthcare system^21^. However, we observed a decrease in contact rates at both GP practices and OOH services for asthma or COPD during the COVID-19 pandemic. In addition to this, the number of urgent contacts did not increase at OOH services. Based on this study, no short-term adverse effects of postponed chronic care for asthma or COPD were apparent. However, there may be long-term consequences because the expected effect of exacerbations due to postponed care in 2020 will only be visible in 2021 and beyond, indicating the need for continued monitoring. In addition, it is possible that postponed GP care may cause an increase in the need for care in other parts of the (acute) health system (i.e., emergency visits, hospital admissions). Further research is needed to assess the impact of postponed chronic care, involving primary care, secondary care, and mortality statistics, and taking into account multiple chronic diseases of patients. If no consequences are observed, the guidelines for disease management for asthma and COPD patients may be reconsidered.

A strength of our study was the inclusion of both GP practices and OOH services, enabling us to examine the impact of the COVID-19 pandemic on care for asthma or COPD for the entire GP care. Another strength was that we used a large data source (routine healthcare data), which ensures the representativeness of the data. The OOH services database covered 70% of the Dutch population and is, therefore, a representative sample of the whole country. The GP practice database consisted of data from the north, east, and south of the Netherlands. However, two of the included regions are regions in which asthma and COPD are more common^37^. Nevertheless, we examined relative differences, where the large population was helpful. For GP practices, we did not include the western region of the Netherlands and, therefore, we lacked data on the metropolitan area. This could potentially affect the findings. However, a Dutch study of healthcare avoidance by patients at the GP and medical specialists during the COVID-19 pandemic (2020) in the metropolitan area showed similar results, i.e. a decrease of 20.2%^38^. A limitation of this study was that we examined the contact rates separately for GP practices and OOH services so that patient-level statements cannot be made about whether postponed care at GP practices resulted in an increase in contact rates at OOH services. Furthermore, our analyses showed that the number of digital consultations was low and unchanged during the COVID-19 pandemic, while other studies showed that GPs in the Netherlands also intensified digital consultations during the first year of the COVID-19 pandemic^39,40^. This is probably due to reimbursement and/or registration bias in the electronic health records data^41^. The means by which contacts are registered or declared may have distorted the proportion of digital consultations in the results. Based on this, we cannot draw any conclusions about the extent of digital consultations for asthma/COPD patients in GP care. In addition, the analysis period may have been too short to observe the effects of postponed GP care for asthma or COPD patients, because the need for more (urgent) care, e.g. due to exacerbations, occurred later. Therefore, future studies should focus on patient care pathways with an extended study period to investigate the consequences of postponed care in GP practices, by linking the data of GP practices, OOH services, and secondary care. Finally, it is important to mention that the incidence of asthma and COPD has decreased in 2020 compared to 2019, which may have resulted in fewer patients with asthma or COPD. This may contribute to the fewer contacts we found in 2020.

In conclusion, the care for patients with asthma and COPD by GPs was greatly impacted by the COVID-19 pandemic, resulting in fewer contacts due to postponed chronic care and fewer exacerbations as a side effect of the COVID-19 measures. This also translated into less high urgent contacts for patients with asthma and COPD with the OOH services. Furthermore, there was a shift towards remote care, which has so far been maintained at OOH services and may also be a tool for efficient asthma and COPD care after the pandemic. This study does not yet show negative effects for patients with asthma or COPD, but it is likely that these are still to come, making it necessary to remain vigilant and continue monitoring in a broader setting, including further research on the long-term impact of the COVID-19 pandemic on care for asthma or COPD patients in primary and secondary care.

## Methods

### Study design and setting

In this observational study, deidentified, routinely recorded, electronic health records data from general practices and OOH services were used. For general practices (during office hours), data from three electronic health records-based repositories in the Netherlands were used: (1) Academic General Practitioner Development Network (Academische Huisartsen Ontwikkel Netwerk—AHON) with 57 participating practices, (2) Family Medicine Network (FaMe-Net) with 6 participating practices, and (3) Research Network Family Medicine Maastricht (RNFM) with 27 participating practices. These are regional networks covering the north, east, and south of the Netherlands. These databases together have a dynamic patient population of ~420,000 patients from the north, south, and east of the Netherlands.

For the OOH services, data from Nivel Primary Care Database (Nivel-PCD), routinely electronic health records from 30 OOH services were used, representing a joint catchment area of almost 12 million people from the Netherlands (60% of all OOH services, and 70% of the Dutch population). The database is representative for the Dutch population concerning sex, age, and region^42^.

### Contact rates and their characteristics

The outcome measures of this study were the contact rates for asthma or COPD, defined by (1) the number of all contacts with the GP or practice nurse per 1000 registered patients in GP practices, and (2) the number of all contacts with OOH services per 1000 inhabitants of OOH services’ catchment area. Contrary to GP practices’ list system, in OOH services there are no patients registered, and therefore, the catchment areas of OOH services were used as the denominator. In both GP practices and OOH services, the diagnoses related to the contacts were recorded routinely with International Classification of Primary Care version 1 (ICPC1 codes). ICPC code R96 was used to identify contacts concerning asthma and R95 for COPD^43–45^. Other outcome measures were the types of contacts and urgency levels (only for OOH services). The types of contacts were derived from reimbursement claims codes and included face-to-face, home visits, and telephone contacts for both GP practices and OOH services, and additionally digital consultation for GP practices. Urgency levels of contacts with OOH services were classified as follows: U0 (resuscitation), U1 (immediate danger to life—immediate care), U2 (threat to vital signs or organ damage—care as soon as possible), U3 (real chance of damage—care within a few hours), U4 (negligible chance of damage—care same day), and U5 (no chance of damage—care next working day).

### Phases of COVID-19 pandemic in the Netherlands

The course of the COVID-19 pandemic, in terms of the number of COVID-19 infections and the related containment measures, varied between various phases of the pandemic. To interpret the changes in contact rates, they must be observed in the context of the pandemic in the Netherlands. Therefore, a brief overview of important containment measures and the waves of COVID-19 infections in 2020 in the Netherlands is provided in Table 5^46^.

**Table 5:** Phases of the COVID-19 pandemic in terms of the containment measures and waves of COVID-19 infections in the Netherlands.

Phases of the COVID-19 pandemic (2020)	Description of containment measures
Phase 0—week 1–8 (non-COVID phase)	- Period before the first COVID-19 infection in the Netherlands.
Phase 1—week 9–24 (phase first wave)	- First wave of COVID-19 infections. - “A lockdown” was introduced (i.e. social distancing, working from home, and the closing of schools, restaurants, museums, sports facilities, and events).
Phase 2—week 25–37 (intermediate phase)	- A calmer period with fewer COVID-19 infections. - The lockdown was abolished, while limited containment measures were retained (i.e. social distancing).
Phase 3—week 38–53 (phase second wave)	- The second wave of COVID-19 infections. - First, a “partial lockdown” was introduced (i.e. social distancing, restaurants closing early, use of facemasks in public spaces, and closing of museums and swimming pools). - Later in this period a “hard lockdown” with extensive containment measures (i.e. closing of schools, non-essential stores, and sports facilities, working from home).

### Data analysis

The characteristics of the population, i.e., the number of contacts for asthma/COPD, and the number of patients with a contact for asthma/COPD are described per 1000 registered patients (GP practices) per year and per 1000 inhabitants of the catchment area (OOH services) per year. In addition, sex, and different age groups are described as the proportion of all contacts for asthma and COPD. All analyses were performed separately for GP practices and OOH services. The contact rates were aggregated and displayed per week for 2019 and 2020. Means and standard deviations were calculated for the contact rates per phase of the COVID-19 pandemic for 2019 and 2020. We performed a sensitivity analysis to investigate whether contact rates should be reported for all registered patients in GP practices or all registered asthma/COPD patients in GP practices. This resulted in no differences, therefore, we described the contacts rate for all registered patients because for OOH services we also plot this against the entire population. Linear regression analysis was performed, with standard errors corrected for autocorrelation of time series (weeks), to investigate the effect of the COVID-19 pandemic (2020) on contact rates for the different phases over time compared to the pre-pandemic period (2019). The types of contacts were shown as the proportional difference between 2020 and 2019 per week. Logistic regressions were performed with standard errors corrected for autocorrelation of time series (weeks), examining the effect of the COVID-19 pandemic on the proportion of the specific types of contacts for the different phases over time between 2019 and 2020. In addition, for the types of contacts, ANOVA with post hoc analyses (Bonferroni) were performed to examine whether there were differences between the phases during the pandemic in 2020. For each urgency level, the number of contacts per 1000 inhabitants of the catchment area and the proportional distribution were calculated. In addition, a two proportions *z*-test was performed to analyse the difference in the urgency levels between phases 1–3 in 2019 and 2020. All analyses were two-tailed and differences were considered statistically significant if the *p* value was lower than 0.05. For the analysis, the software programme STATA was used (version 16.1).

### Ethical considerations

Ethical approval for this study was waived by the medical ethics committee of the University Medical Centre Groningen (reference number: 2020/309). The use of electronic health record data is permitted under certain conditions by Dutch law both for the data from the three general practice registration networks and Nivel-PCD. According to this legislation, neither obtaining informed consent from patients nor approval by a medical ethics committee is obligatory for these types of observational studies, containing no directly identifiable patient data (art. 24 GDPR Implementation Act jo art. 9.2 sub j GDPR). For Nivel-PCD, the project has been approved by the relevant governance bodies of Nivel-PCD under no. NZR-00320.087.

### Reporting summary

Further information on research design is available in the Nature Research Reporting Summary linked to this article.

## Supplementary information

Supplementary Material

Reporting Summary

## Data Availability

The data underlying this article (both for the three general practice registration networks and Nivel-PCD) will be shared at reasonable request to the corresponding author. For Nivel-PCD this follows the governance of the “Nivel Primary Care Database”. Data in the “Nivel Primary Care Database” are extracted from the electronic health records of OOH services. The use of the data for research purposes is subject to approval by a committee representing the health professionals who recorded the data in their electronic health records, reviewing proposals on the relevance for, and privacy of, the OOH services and their patients (https://www.nivel.nl/en/nivel-zorgregistraties-eerste-lijn/nivel-primary-care-database).
