# Supplementary material for: Care by general practitioners for patients with asthma or COPD during the COVID-19 pandemic
Source: NPJ Prim Care Respir Med. 2023 Apr 8;33:15. doi: 10.1038/s41533-023-00340-z (PMC10082338; doi:10.1038/s41533-023-00340-z)
Supplement: Supplementary file 1 — Supplementary Material [file 41533_2023_340_MOESM1_ESM.pdf]

Supplementary files

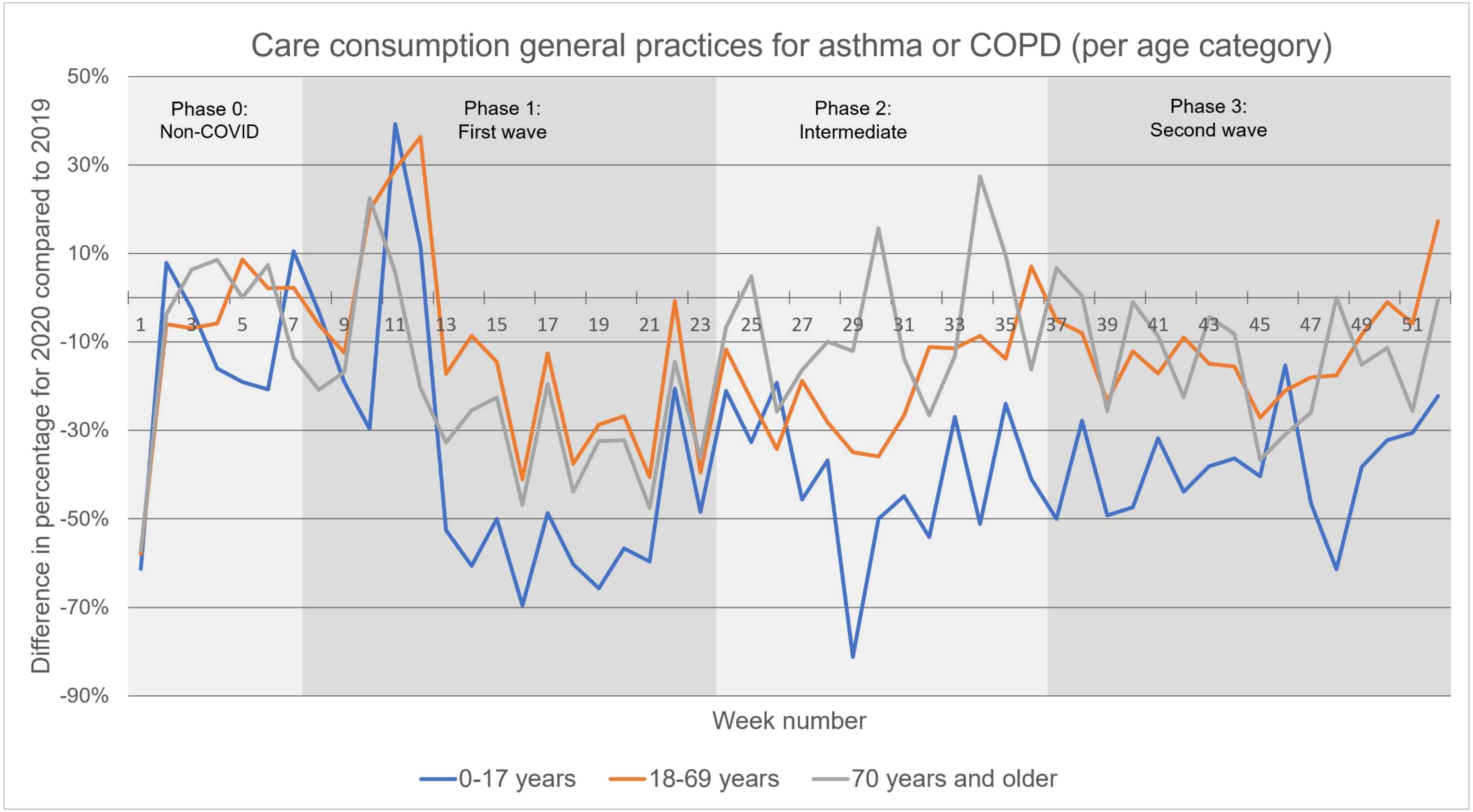

Supplementary Figure 1. The difference in care consumption in GP practices for asthma or COPD, 2020 compared to 2019 – per age category.

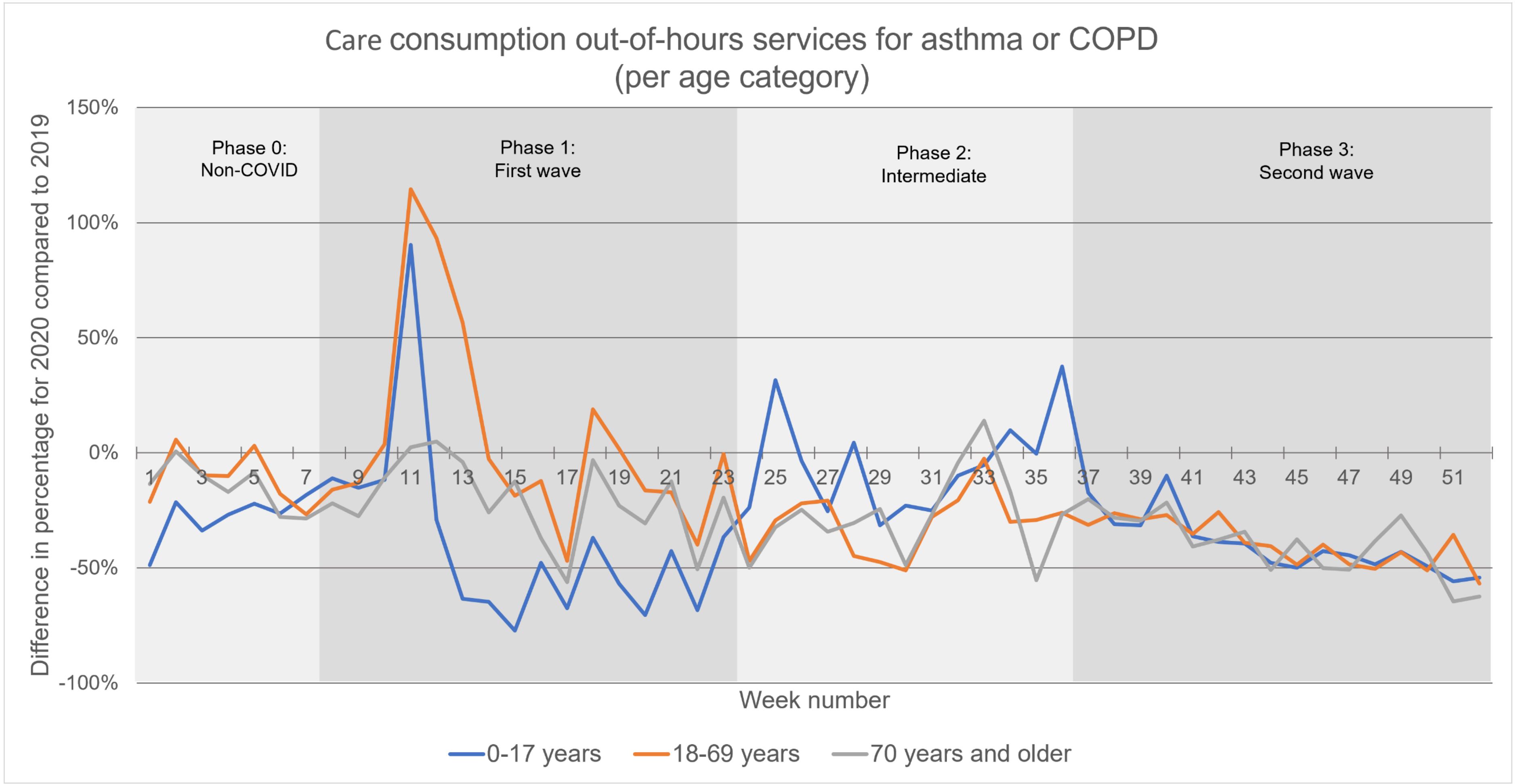

Supplementary Figure 2. The difference in care consumption at OOH services for asthma or COPD, 2020 compared to 2019 – per age category.
