# Supplementary material for: Care by general practitioners for patients with asthma or COPD during the COVID-19 pandemic
Source: NPJ Prim Care Respir Med. 2023 Apr 8;33:15. doi: 10.1038/s41533-023-00340-z (PMC10082338; doi:10.1038/s41533-023-00340-z)
Supplement: Supplementary file 2 — Reporting Summary [file 41533_2023_340_MOESM2_ESM.pdf]

nature portfolio

Corresponding author(s): CJ Rijpkema

Last updated by author(s): Feb 17, 2023

Reporting Summary

Nature Portfolio wishes to improve the reproducibility of the work that we publish. This form provides structure for consistency and transparency in reporting. For further information on Nature Portfolio policies, see our Editorial Policies and the Editorial Policy Checklist.

Statistics

For all statistical analyses, confirm that the following items are present in the figure legend, table legend, main text, or Methods section.

n/a	Confirmed	
☐	☒	The exact sample size (*n*) for each experimental group/condition, given as a discrete number and unit of measurement
☒	☐	A statement on whether measurements were taken from distinct samples or whether the same sample was measured repeatedly
☐	☒	The statistical test(s) used AND whether they are one- or two-sided<br>*Only common tests should be described solely by name; describe more complex techniques in the Methods section.*
☐	☒	A description of all covariates tested
☐	☒	A description of any assumptions or corrections, such as tests of normality and adjustment for multiple comparisons
☐	☒	A full description of the statistical parameters including central tendency (e.g. means) or other basic estimates (e.g. regression coefficient) AND variation (e.g. standard deviation) or associated estimates of uncertainty (e.g. confidence intervals)
☐	☒	For null hypothesis testing, the test statistic (e.g. *F*, *t*, *r*) with confidence intervals, effect sizes, degrees of freedom and *P* value noted<br>*Give P values as exact values whenever suitable.*
☒	☐	For Bayesian analysis, information on the choice of priors and Markov chain Monte Carlo settings
☒	☐	For hierarchical and complex designs, identification of the appropriate level for tests and full reporting of outcomes
☒	☐	Estimates of effect sizes (e.g. Cohen's *d*, Pearson's *r*), indicating how they were calculated

Our web collection on statistics for biologists contains articles on many of the points above.

Software and code

Policy information about availability of computer code

Data collection	NA
Data analysis	NA

For manuscripts utilizing custom algorithms or software that are central to the research but not yet described in published literature, software must be made available to editors and reviewers. We strongly encourage code deposition in a community repository (e.g. GitHub). See the Nature Portfolio guidelines for submitting code & software for further information.

Data

Policy information about availability of data

All manuscripts must include a data availability statement. This statement should provide the following information, where applicable:

- Accession codes, unique identifiers, or web links for publicly available datasets
- A description of any restrictions on data availability
- For clinical datasets or third party data, please ensure that the statement adheres to our policy

The data underlying this article (both for the three general practice registration networks and Nivel-PCD) will be shared at reasonable request to the corresponding author. For Nivel-PCD this follows the governance of the 'Nivel Primary Care Database'. Data in the 'Nivel Primary Care Database' are extracted from the electronic health records of OOH services. The use of the data for research purposes is subject to approval by a committee representing the health professionals who

recorded the data in their electronic health records, reviewing proposals on the relevance for, and privacy of, the OOH services and their patients. (https://www.nivel.nl/en/nivel-zorgregistraties-eerste-lijn/nivel-primary-care-database).

Human research participants

Policy information about studies involving human research participants and Sex and Gender in Research.

Reporting on sex and gender	The findings did not only apply to one sex. We used the sex variable from the general practice registry and out-of-hours services registry. We did not report on an individual level. In Table 2 we have given a descriptive representation of how many patients are male and female in the study population (in percentage).
Population characteristics	The population characteristics are displayed in Table 2.
Recruitment	In our observational study, deidentified, routinely recorded, electronic health records data from general practices and out-of-hours services were used. For general practices (during office hours), data from three electronic health records-based repositories in the Netherlands were used: 1) Academic General Practitioner Development Network (Academische Huisartsen Ontwikkel Netwerk – AHON) with 57 participating practices, 2) Family Medicine Network (FaMe-Net) with 6 participating practices, and 3) Research Network Family Medicine Maastricht (RNFM) with 27 participating practices. For the OOH services, data from Nivel Primary Care Database (Nivel-PCD) were used, representing a joint catchment area of almost 12 million people from the Netherlands (60% of all OOH services, and 70% of the Dutch population).
Ethics oversight	Ethical approval for this study was waived by the medical ethics committee of the University Medical Centre Groningen (reference number: 2020/309). For Nivel-PCD, the project has been approved by the relevant governance bodies of Nivel-PCD under no. NZR-00320.087.

Note that full information on the approval of the study protocol must also be provided in the manuscript.

Field-specific reporting

Please select the one below that is the best fit for your research. If you are not sure, read the appropriate sections before making your selection.

☒ Life sciences ☐ Behavioural & social sciences ☐ Ecological, evolutionary & environmental sciences

For a reference copy of the document with all sections, see nature.com/documents/nr-reporting-summary-flat.pdf

Life sciences study design

All studies must disclose on these points even when the disclosure is negative.

Sample size	No sample-size calculations were performed, because our databases consists of a sufficient number of patients (i.e. 420.000 patients for general practices and 70% of the Dutch population for out-of-hours services)
Data exclusions	The data specialists prepared the data based on quality criteria and excluded general practices/out-of-hours services when they do not meet the criteria. Researchers excluded contacts who were not for asthma or COPD.
Replication	When (other) researchers have access to the data, analyses can be reproduced.
Randomization	Not relevant to our study
Blinding	Blinding was not relevent to our study

Reporting for specific materials, systems and methods

We require information from authors about some types of materials, experimental systems and methods used in many studies. Here, indicate whether each material, system or method listed is relevant to your study. If you are not sure if a list item applies to your research, read the appropriate section before selecting a response.

Materials & experimental systems

n/a	Involved in the study
☒	☐ Antibodies
☒	☐ Eukaryotic cell lines
☒	☐ Palaeontology and archaeology
☒	☐ Animals and other organisms
☒	☐ Clinical data
☒	☐ Dual use research of concern

Methods

n/a	Involved in the study
☒	☐ ChIP-seq
☒	☐ Flow cytometry
☒	☐ MRI-based neuroimaging
